# A New Therapeutic Strategy for Recurrent Ovarian Cancer―Bevacizumab beyond Progressive Disease

**DOI:** 10.3390/healthcare7030109

**Published:** 2019-09-19

**Authors:** Tadahiro Shoji, Hisashi Eto, Takanori Sato, Rikako Soma, Daisuke Fukagawa, Hidetoshi Tomabechi, Eriko Takatori, Takayuki Nagasawa, Seiya Sato, Masahiro Kagabu, Tsukasa Baba

**Affiliations:** Department Obstetrics and Gynecology, Iwate Medical University School of Medicine, 19-1 Uchimaru, Morioka, Iwate 020-8505, Japan; etohisa@iwate-med.ac.jp (H.E.); somar@iwate-med.ac.jp (R.S.); dfukaga@yahoo.co.jp (D.F.); tbechi@iwate-med.ac.jp (H.T.); sppe8459@yahoo.co.jp (E.T.); shirokuma723@ybb.ne.jp (T.N.); sato_seiya9534@yahoo.co.jp (S.S.); m.kagabu@nifty.com (M.K.); babatsu@iwate-med.ac.jp (T.B.)

**Keywords:** recurrent ovarian cancer, chemotherapy, bevacizumab beyond PD, MITO16/MaNGO-OV2B, JGOG3023

## Abstract

Treatment beyond progressive disease (PD) is a concept that even after drugs become ineffective, their continued use is more beneficial for patients than their discontinuation. In recent years, a concept of bevacizumab beyond PD (BBP) has attracted attention in the treatment of various cancers, and the usefulness of this concept has been evaluated. BBP has been proven to prolong overall survival (OS) in recurrent colorectal cancer and progression-free survival (PFS) in recurrent breast and lung cancers. With regard to the treatment of ovarian cancer, the MITO16/MaNGO-OV2B study (the Multicenter Phase III Randomized Study with Second Line Chemotherapy Plus or Minus Bevacizumab in Patients with Platinum Sensitive Epithelial Ovarian Cancer Recurrence After a Bevacizumab/Chemotherapy First Line) was conducted in patients with platinum-sensitive recurrence and the JGOG3023 study (the Open-Label, Randomized, Phase II Trial Evaluating the Efficacy and Safety of Standard of Care with or Without Bevacizumab in Platinum-Resistant Ovarian Cancer Patients Previously Treated with Bevacizumab for Front-Line or Platinum-Sensitive Ovarian Cancer) was conducted in patients with platinum-resistant recurrence. The MITO16/MaNGO-OV2B study, reported in the 2018 annual meeting of the American Society of Clinical Oncology, showed that BBP achieved prolonged PFS. In the JGOG3023 study, enrollment of patients was completed in December 2018, and the follow-up period has been initiated. Proving the effectiveness of BBP in the treatment of ovarian cancer may provide a new therapeutic strategy and contribute to improved treatment outcomes in patients with poor prognosis and limited therapeutic options.

## 1. Introduction

In recent years, not only vascular endothelial growth factors (VEGFs), but also basic fibroblast growth factors (bFGFs) and transforming growth factor-β (TGF-β) have been shown to be involved in tumor proliferation [1,2,3]. Bevacizumab, a monoclonal antibody to VEGF, is a molecular targeted drug that binds to VEGFs to inhibit their activity, suppress angiogenesis, and prevent tumor proliferation and metastasis.

In Japan, bevacizumab was listed on the National Health Insurance Price List for the treatment of ovarian cancer in November 2013. At present, the drug is widely used in not only first-line therapy but also the treatment of recurrent ovarian cancer. Additionally, bevacizumab is widely used to treat other types of cancers. It would not be an overstatement to say that all types of cancers are currently treated with bevacizumab [4,5,6,7,8].

In recent years, a concept of bevacizumab beyond progressive disease (BBP) has attracted attention in the treatment of various cancers; its usefulness has been demonstrated. BBP has also been evaluated for patients with ovarian cancer in Japan and other countries and proven useful. In this article, we provide an overview of BBP.

## 2. Concept of Treatment beyond Progressive Disease

The continued use of a drug even after patients developed progressive disease (PD) is referred to as “treatment beyond PD” or “treatment beyond progression.” It implies that even after a drug becomes ineffective, its continued use is more beneficial for patients than its discontinuation. Randomized controlled trials have recently reported that when patients developed PD after multi-agent combination chemotherapy, combination chemotherapy with the continued use of some of the same drugs is more useful than that with a completely different set of drugs.

In actual clinical practice, the effects of chemotherapy are generally determined by the Response Evaluation Criteria in Solid Tumors (RECIST) [9]. However, the RECIST was developed to objectively assess changes in the size of solid tumors and was not intended to be used for making decisions on whether treatment should be continued in individual patients. In other words, when patients are determined to develop PD by the RECIST, automatic discontinuation or modification of treatment does not necessarily contribute to the prolongation of survival or maintenance/improvement of the quality of life of the patients.

## 3. Clinical Studies Validating the Usefulness of BBP

Clinical studies have proven the usefulness of BBP only for the treatment of colorectal, breast, and lung cancers. The contents and results of these clinical studies are summarized in Table 1 and briefly described in the following subsections.

### 3.1. BRiTE: The Bevacizumab Regimens’ Investigation of Treatment Effects

The BRiTE study included 1445 patients with unresectable advanced/recurrent colorectal cancer that progressed during first-line therapy, including bevacizumab. They were divided into three groups: the untreated group in which 253 patients were untreated after progression, the non-BBP group in which 531 patients were treated without bevacizumab after progression, and the BBP group in which 642 patients continued to receive bevacizumab after progression. The median overall survival (OS) after the start of the first-line therapy was 12.6 months in the untreated group, 19.9 months in the non-BBP group, and 31.8 months in the BBP group. Multivariate analysis, comparing the non-BBP and BBP groups, showed that BBP was an independent factor for prolonged OS (hazard ratio (HR) = 0.49; *p* < 0.001). This study was the first to demonstrate the usefulness of BBP. However, it also had limitations. For example, patients with an Eastern Cooperative Oncology Group performance status of 0 accounted for 50% in the BBP group and 40% in the non-BBP group. An anti-epidermal growth factor receptor (EGFR) monoclonal antibody was used in 50.9% of the patients in the non-BBP group. The BBP group did not include any patients with rapid progression during first-line therapy with bevacizumab [10]. However this trial was a cohort study, the randomized controlled trial was planned at the later date.

### 3.2. ARIES: The Avastin Registry—Investigation of Effectiveness and Safety (ARIES)

This observational study aimed to assess postprogression survival (PPS) in patients with unresectable advanced/recurrent colorectal cancer who started treatment with and without bevacizumab within 2 months after confirmed progression (BBP and non-BBP groups). As with the BRiTE study, the ARIES study showed that the median PPS in the BBP group was 14.4 months and significantly longer than that of 10.6 months observed in the non-BBP group (*p* = 0.02; HR = 0.84). This study also reported that SBP after first-line therapy is positively correlated with cumulative exposure to bevacizumab [11].

### 3.3. ML18147: A Randomized Open-Label Phase III Intergroup Study, Effect of Adding Bevacizumab to Cross Over Fluoropyrimidine Based Chemotherapy (CTx) in Patients with Metastatic Colorectal Cancer and Disease Progression under First-Line Standard CTx/Bevacizumab Combination

This study included patients who had been treated with bevacizumab plus chemotherapy (irinotecan-based or oxaliplatin-based) as first-line therapy and become unresponsive to the treatment. They were divided into two groups for comparison: the BBP group in which 409 patients were treated with standard chemotherapy (irinotecan-based or oxaliplatin-based) with bevacizumab as second-line therapy and the non-BBP group in which 410 patients were treated with the same chemotherapy without bevacizumab. The median OS in the BBP group was 11.2 months and significantly longer than that of 9.8 months observed in the non-BBP group (*p* = 0.0062; HR = 0.81). Furthermore, the median progression-free survival (PFS) was 5.7 months in the BBP group and 4.1 months in the non-BBP group (*p* < 0.0001: HR = 0.68) [12]. This prospective study proved the usefulness of BBP for the first time.

### 3.4. TANIA: The Study of Avastin (Bevacizumab) in Combination with Chemotherapy in Patients with Breast Cancer Progressing after First-Line Therapy with Chemotherapy

This study included 494 patients treated with chemotherapy plus bevacizumab as first-line therapy for human epidermal growth factor receptor type 2 (HER2)-negative, locally recurrent/metastatic breast cancer. They were randomly assigned to continue chemotherapy with bevacizumab (BBP group) or without bevacizumab (non-BBP group) as second-line and subsequent therapies. In the non-BBP group, patients experiencing PD during the second-line therapy were treated with chemotherapy without bevacizumab as the third-line therapy. No crossover was allowed. The median PFS in the BBP group was 6.3 months and significantly longer than that of 4.2 months observed in the non-BBP group (*p* = 0.0068; HR = 0.75) [13].

### 3.5. WJOG5910L: The Open-Label, Randomized, Phase IIIb Trial Evaluating the Efficacy and Safety of Standard of Care +/− Continuous Bevacizumab Treatment beyond Progression of Disease (PD) in Patients with Advanced Non-Squamous Non-Small Cell Lung Cancer (NSCLC) after First-Line Treatment with Bevacizumab Plus a Platinum Doublet-Containing Chemotherapy Including 100 Patients with Advanced Non-Squamous Non-Small Cell Lung Cancer Who Received Platinum-Based Chemotherapy Plus Bevacizumab and Experienced Disease Progression

This study compared docetaxel monotherapy and a combination of docetaxel and bevacizumab. The primary endpoint was PFS, and the secondary endpoints were OS, response rate, and safety. Among patients positive for EGFR mutations, those who had received first-line therapy with EGFR-tyrosine kinase inhibitor before platinum-based anticancer drugs were also allowed to be enrolled. The median PFS was 4.4 months in the combination group and 3.4 in the monotherapy group. The PFS in the combination group was better, with an HR of 0.71 (95% confidence interval (CI): 0.46–1.19) and a stratified *p*-value of 0.058 (one-sided). The median OS was 13.1 months in the combination group and 11.0 months in the monotherapy group. The OS in the combination group was also better, with an HR of 0.74 (95% CI: 0.46–1.19) and a stratified *p*-value of 0.11 (one-sided). The response rates were 36% (95% CI: 22.9–50.8) in the combination group and 26% (95% CI: 14.6–40.3) in the monotherapy group. The response rates also tended to be better in the combination group (*p* = 0.387) [14].

## 4. BBP for Ovarian Cancer

Bevacizumab was proven to be a useful drug for first-line chemotherapy of ovarian cancer by the GOG0218 (the Placebo, Versus Carboplatin and Paclitaxel Plus Concurrent and Extended Bevacizumab, in Women with Newly Diagnosed, Previously Untreated, Stage III or IV Epithelial Ovarian, Primary Peritoneal or Fallopian Tube Cancer) and ICON7 (the Randomized, Two-Arm, Multi-Center Gynecologic Cancer Intergroup Trial of Adding Bevacizumab to Standard Chemotherapy (Carboplatin and Paclitaxel) in Patients with Epithelial Ovarian Cancer) [15,16]. In Japan, bevacizumab was listed on the National Health Insurance Price List for the treatment of ovarian cancer in November 2013. At present, facilities using the drug for first-line chemotherapy are increasing. However, it is difficult to prevent recurrence in patients with advanced ovarian cancer. Many of them eventually develop platinum-sensitive or platinum-resistant recurrence. Bevacizumab has been proven useful for treatment of patients with platinum-sensitive recurrence by the OCEANS (the Randomized, Double-Blind, Placebo-Controlled Phase III Trial of Chemotherapy With or Without Bevacizumab in Patients with Platinum-Sensitive Recurrent Epithelial Ovarian, Primary Peritoneal, or Fallopian Tube Cancer) [17] and for treatment of patients with platinum-resistant recurrence by the AURELIA (the Randomized Phase III Trial Evaluating Bevacizumab Combined with Chemotherapy for Platinum-Resistant Recurrent Ovarian Cancer) [18]. However, the usefulness of BBP for the treatment of patients with recurrent ovarian cancer has not yet been proven. Studies on ovarian cancer lag behind studies on other types of cancers. Nevertheless, the MITO16/MaNGO-OV2B study (the Multicenter Phase III Randomized Study with Second Line Chemotherapy Plus or Minus Bevacizumab in Patients with Platinum Sensitive Epithelial Ovarian Cancer Recurrence After a Bevacizumab/Chemotherapy First Line) was conducted in patients with platinum-sensitive recurrence. The JGOG3023 study (the Open-label, Randomized, Phase II Trial Evaluating the Efficacy and Safety of Standard of Care With or Without Bevacizumab in Platinum-Resistant Ovarian Cancer Patients Previously Treated with Bevacizumab for Front-Line or Platinum-Sensitive Ovarian Cancer) is in progress and is evaluating in patients with platinum-resistant recurrence. We here briefly describe the results of the MITO16/MaNGO-OV2B study that were presented in the 2018 annual meeting of the American Society of Clinical Oncology (ASCO), and the JGOG3023 study, which has transitioned from the enrollment period to the follow-up period.

### 4.1. MITO16/MaNGO-OV2B Study

This study included 405 patients with platinum-sensitive recurrent ovarian cancer who were previously treated with bevacizumab. The patients in the control arm received carboplatin-based chemotherapy combined with one drug selected from doxil, gemcitabine, and paclitaxel by the attending physicians. In doxil + carboplatin therapy, both doxil at 30 mg/m^2^ and carboplatin at an area under the curve (AUC) of 5 were administered on day 1 every 4 weeks. In gemcitabine + carboplatin therapy, gemcitabine at 1000 mg/m^2^ was administered on days 1 and 8 every 3 weeks and carboplatin at an AUC of 4 was administered on day 1 every 3 weeks. In paclitaxel + carboplatin therapy, both paclitaxel at 175 mg/m^2^ and carboplatin at an AUC of 5 were administered on day 1 every 3 weeks. In contrast, in the bevacizumab-combination chemotherapy group, bevacizumab was administered at 10 mg/kg every 2 weeks to patients receiving doxil + carboplatin therapy and at 15 mg/kg every 3 weeks to patients receiving gemcitabine + carboplatin or paclitaxel + carboplatin therapy. The primary endpoint was PFS and the secondary endpoints were OS and adverse events. When the median follow-up period reached 20.3 months, the median PFS was 8.8 months in the chemotherapy group and 11.8 months in the bevacizumab-combination chemotherapy group. In the latter group, PFS was significantly longer with an HR of 0.51 (95% CI: 0.41–0.65) and a *p*-value of less than 0.001. Meanwhile, the median OS was 27.1 months in the chemotherapy group and 26.6 months in the bevacizumab-combination chemotherapy group, showing no significant difference (HR: 1.00; 95% CI: 0.73–1.39; *p* = 0.98). Grade 3 or greater adverse events with a significant difference between the chemotherapy and bevacizumab-combination chemotherapy groups were hypertension (0% vs. 28.9%, *p* < 0.001), proteinuria (0% vs. 3.9%, *p* = 0.007), and thrombocytopenia (21.5% vs. 30.3%, *p* = 0.04). However, the results of the subgroup analysis are more interesting. Because paclitaxel + carboplatin therapy had been administered in a previous treatment, this therapy was avoided, and either doxil + carboplatin or gemcitabine + carboplatin therapy was administered to 363 of the 405 patients (89.6%). However, the longest PFS was observed in the patients receiving paclitaxel + carboplatin therapy combined with bevacizumab [19]. This result appeared to provide renewed evidence that paclitaxel is a key drug in the treatment of ovarian cancer.

### 4.2. JGOG3023 Study

**Figure 1:**
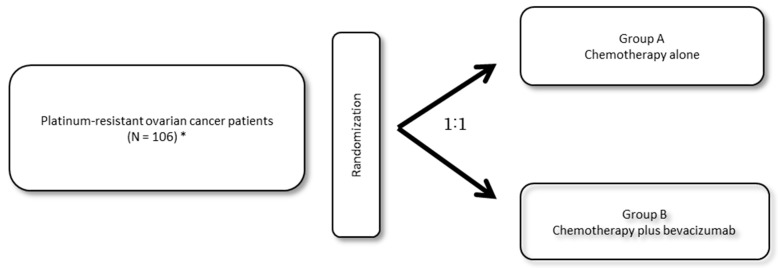
JGOG3023 Schema. Platinum-resistant ovarian cancer patients will be randomized 1:1 to treatment with chemotherapy alone or chemotherapy plus bevacizumab. *Defined as progression within < 6 months from completion of a minimum of three platinum therapy (including bevacizumab) cycles.

Platinum-resistant recurrence is considered to be associated with poor prognosis. The 2015 version of the Japanese guidelines for the treatment of ovarian cancer states that single-agent chemotherapy, radiotherapy for symptomatic relief, and best supportive care should be considered for patients with this condition. An exploration of new therapeutic options for such patients with poor prognosis is essential for improving the prognosis of ovarian cancer. This study began in June 2015 as part of the clinical trials conducted by the Japanese Gynecologic Oncology Group (JGOG) (Figure 1). Including patients with platinum-resistant recurrent ovarian cancer who were previously treated with bevacizumab, this study aims to assess the efficacy and safety of combination chemotherapy with bevacizumab in comparison with single-agent chemotherapy [20]. The subjects of this study are patients who received three cycles or more of bevacizumab + platinum-based combination chemotherapy and experienced recurrence or progression during chemotherapy or within less than 6 months from the last day of administration of platinum agents. The primary endpoint is PFS and the secondary endpoints are safety, objective response rate, OS, number of paracentesis, and response rate based on CA125. The administered chemotherapeutic regimens are as follows: In the single-agent chemotherapy group, patients are treated with doxil alone administered at 40 or 50 mg/m^2^ on day 1 every 4 weeks, nogitecan alone administered at 1.25 mg/m^2^ on days 1 to 5 every 3 weeks, paclitaxel alone administered at 80 mg/m^2^ on days 1, 8, and 15 every 3 weeks, or gemcitabine alone administered at 1000 mg/m^2^ on days 1 and 8 every 3 weeks. From these chemotherapeutic regimens, attending physicians are allowed to select one to be administered. In the bevacizumab-combination group, bevacizumab is administered at 15 mg/kg every 3 weeks in addition to the above-described chemotherapeutic regimens. All patients are to be treated until disease progression. The AVF2949g study, a single-arm phase II clinical study of bevacizumab monotherapy in patients with recurrent ovarian cancer (including both platinum-sensitive and platinum-resistant recurrent ovarian cancer), was discontinued because five of the 44 accumulated patients developed gastrointestinal perforation (GIP). Those five patients had received prior treatment with three regimens, and therefore, cautious use of bevacizumab is warranted in patients heavily pretreated with bevacizumab. Gastrointestinal infiltration that results from ovarian cancer metastasis was observed in 40 of 44 patients included in the study [21]. In JGOG3023 study the patients will be excluded based on the following criteria to reduce the GIP risk: with ≥ 4 previous anticancer regimens; with a history of bowel obstruction, including subocclusive disease, related to the underlying disease and history of abdominal fistula, gastrointestinal perforation, or intra-abdominal abscess; with evidence of rectosigmoid involvement by pelvic examination or bowel involvement on computed tomography or clinical symptoms of bowel obstruction.

As of December 2018, 103 patients were enrolled and follow-up has been initiated. The JGOG3023 study is expected to establish new therapeutic strategies for platinum-resistant recurrent ovarian cancer. Simultaneously, this clinical study may contribute to an improved prognosis of ovarian cancer. In addition, we consider that presenting data from Japan to the world has great significance.

## 5. Is Paclitaxel a Potential Key Anticancer Drug Used in Combination with Bevacizumab?

Both the MITO16/MaNGO-OV2B and JGOG3023 studies allow attending physicians to select a chemotherapeutic regimen to be administered at their discretion. However, treatment outcomes have been reported to vary, depending on selected chemotherapeutic regimens. In this section, we discuss which anticancer drug is suitable to be used in combination with bevacizumab.

For a combination of single-agent chemotherapy and bevacizumab, scientific evidence is provided by the AURELIA study, which is a randomized phase III clinical study that compared single-agent chemotherapy alone and single-agent chemotherapy plus bevacizumab in patients who experienced PD within 6 months after the completion of four cycles or more of platinum-based chemotherapy. Patients who had been treated with bevacizumab in previous treatment were also allowed to be included. For single-agent chemotherapy, the investigators selected one of three drugs: doxil, nogitecan, and paclitaxel. The median PFS, the primary endpoint, was 3.4 months in the single-agent chemotherapy group and 6.7 months in the bevacizumab-combination group. The PFS in the latter group was significantly better, with an HR of 0.48 (95% CI: 0.38–0.60) and a P-value of less than 0.001. Based on subanalysis stratified by chemotherapeutic regimen, the median PFS was 5.4 months in the doxil + bevacizumab group and 5.8 months in the nogitecan + bevacizumab group, and the response rates were 18.3% and 22.8%, respectively. In contrast, the paclitaxel + bevacizumab group showed a median PFS of 10.4 months and a response rate of 51.7%, which were improbably better results for patients with platinum-resistant recurrence [18].

Next, a combination of multi-agent chemotherapy and bevacizumab is discussed. Because the data of the MITO16/MaNGO-OV2B study have not yet been published, our discussion is based on the data presented at the 2018 annual meeting of the ASCO. The HRs were 0.41 (95% CI: 0.28–0.60) for doxil + carboplatin therapy plus bevacizumab and 0.59 (95% CI: 0.42–0.83) for gemcitabine + carboplatin therapy plus bevacizumab. In contrast, paclitaxel + carboplatin therapy plus bevacizumab showed better treatment outcomes with an HR of 0.34 (95% CI: 0.15–0.80) [19].

In patients with platinum-sensitive or platinum-resistant recurrence, recurrence is presumably associated with previous treatment with paclitaxel. Physicians may be reluctant to administer paclitaxel to such patients again. It is important to note the chemotherapy regimens at AURELIA study and MITO16/MaNGO-OV2B were not randomized and were determined by the attending physicians, however, the results of the these studies suggest that better treatment outcomes can be achieved when paclitaxel is combined with bevacizumab. We consider that when bevacizumab is concurrently used for patients with peripheral neuropathy of grade 1 or below caused by paclitaxel, paclitaxel should be aggressively selected; whether this approach is beneficial will be determined in future reports.

## 6. Conclusions

In this article, we have provided an overview of the usefulness of BBP, with a primary focus on ovarian cancer as well as other types of cancers. BBP may be proven to be a novel and useful therapeutic strategy for recurrent ovarian cancer. At present, in Japan, the JGOG3023 study is in progress to validate new therapeutic strategies for recurrent ovarian cancer. If BBP is proven useful for the treatment of platinum-resistant recurrent ovarian cancer, the prognosis of this condition, which is considered to be poor, could be improved. Furthermore, BBP might also prove to be an ideal alternative treatment for patients with limited therapeutic options and could be included in the guidelines for the treatment of ovarian cancer, not only in Japan but also globally. With this Japanese study, we hope to provide enough evidence and establish the advantages of BBP for the treatment of platinum-resistant recurrence. We also hope that this article will assist and improve the knowledge of young physicians aspiring to become obstetricians or gynecological oncologists.

## Figures and Tables

**Table 1:** Previous Clinical Trials of Bevacizumab beyond PD.

Trial	Design	Cancer Type	Primary Chemotherapy	Regimens	Patients	Primary Endpoint	Results	*p* Value
BRiTE^10)^	cohort	Colorectal cancer	CT+BEV	CT (control) CT+BEV Observation	531 642 253	OS	19.9m 31.8m 12.6m	*p* < 0.001
ARIES^11)^	cohort	Colorectal cancer	CT+BEV	CT (control) CT+BEV	667 438	PPS	10.6m 14.4m	*p* = 0.02
ML18147^12)^	PIII	Colorectal cancer	CT+BEV	CT (control) CT+BEV	410 409	OS	9.8m 11.2m	*p* = 0.0062
TANIA^13)^	PIII	Brest cancer	CT+BEV	CT(control) CT+BEV	247 247	PFS (2^nd^ line)	4.2m 6.3m	*p* = 0.0068
WJOG5910L^14)^	PII	Lung cancer	CT+BEV	CT(control) CT+BEV	50 50	PFS	3.4m 4.4m	*p* = 0.058

OS: overall survival, CT: chemotherapy, BEV: bevacizumab, PPS: postprogression survival, PFS: progression free survival, m: months.
